# Radiological DESH score cannot predict outcome after shunt surgery: A Swedish prospective follow-up study

**DOI:** 10.1177/19714009261494951

**Published:** 2026-10-07

**Authors:** Clara Constantinescu, Mia Emgård, Karin Sundström, Dan Farahmand, Mats Tullberg, Doerthe Ziegelitz, Per Hellström

**Affiliations:** 1 Hydrocephalus Research Unit, Department of Clinical Neuroscience, Institute of Neuroscience and Physiology, Sahlgrenska Academy, 195564 University of Gothenburg, Gothenburg, Sweden; 2 Department of Neuropsychiatry, Sahlgrenska University Hospital, Region Västra Götaland, Gothenburg, Sweden; 3 Department of Radiology, Institute of Clinical Sciences, Sahlgrenska Academy, 195564 University of Gothenburg, Gothenburg, Sweden; 4 Department of Radiology, 3572 Sahlgrenska University Hospital, Region Västra Götaland, Gothenburg, Sweden; 5 Department of Neurology, 70712 Sahlgrenska University Hospital, Gothenburg, Sweden

**Keywords:** idiopathic normal pressure hydrocephalus, MRI, imaging markers, disproportionately enlarged subarachnoid-space hydrocephalus, DESH score

## Abstract

**Background:**

Disproportionately enlarged subarachnoid-space hydrocephalus (DESH) has been proposed as a radiological predictor of shunt responsiveness in idiopathic normal pressure hydrocephalus (iNPH), but previous studies have reported inconsistent results. We investigated the association between preoperative DESH score and postoperative outcome after shunt surgery in a prospective iNPH cohort.

**Methods:**

This prospective study included 101 consecutive patients with iNPH from the Gothenburg Prediction of Outcome in iNPH (POiNT) study. Preoperative DESH scores were assessed on MRI using previously published criteria. Clinical outcomes included the Gothenburg iNPH scale, modified Rankin Scale (mRS), Mini-Mental State Examination (MMSE), and Timed Up and Go test (TUG-t). The primary outcome was improvement of ≥5 points on the iNPH scale.

**Results:**

Mean preoperative DESH score was 6.3 ± 1.7. At follow-up, the mean iNPH scale score improved from 50.8 ± 19.8 to 63.3 ± 20.0 (*p* < .001), with 70% improving by ≥5 points. Preoperative DESH score was not associated with postoperative change in iNPH scale score (*r* = −0.123, *p* = .219) or with responder status for any outcome. In multivariable analysis, DESH score was not independently associated with postoperative iNPH scale score after adjustment for baseline iNPH scale score, age, and disease duration. DESH score and Evans’ index decreased and callosal angle increased after surgery (all *p* < .001).

**Conclusions:**

Preoperative DESH score was not associated with postoperative clinical improvement after shunt surgery in patients with iNPH. Although DESH-related imaging markers changed significantly after surgery, baseline DESH score had limited value as an independent predictor of shunt responsiveness.

## Background

Idiopathic normal pressure hydrocephalus (iNPH) is the most common form of adult hydrocephalus, with symptom onset typically occurring around 70 years of age. In a Swedish population-based cohort, the prevalence of iNPH was reported to be 2.9% among 75-year-olds.^1^ Shunt surgery is currently the only effective treatment and may improve symptoms in up to 80% of patients.^2,3^ However, not all patients respond to surgery, and shunt treatment carries a risk of complications. Identifying reliable predictors of postoperative outcome therefore remains an important clinical challenge.

Several supplementary tests, including the tap test and infusion test, have been proposed to predict shunt responsiveness, but their negative predictive value is limited. Certain radiological changes represent hallmarks of iNPH and numerous radiological markers and composite imaging scales have therefore been investigated as potential predictors of postoperative improvement.^4–12^

One such imaging marker is the disproportionately enlarged subarachnoid-space hydrocephalus (DESH) score, originally described by Shinoda et al.^4^ The DESH score is based on characteristic MRI findings, defined as a combination of ventriculomegaly, tight high convexity sulci and Sylvian fissure dilation in iNPH, and has been proposed as a radiological predictor of shunt response.^4^ Several studies have reported associations between higher preoperative DESH scores and favorable postoperative outcome after shunt surgery,^4–7^ whereas others have questioned its predictive value.^8,9^ A recent systematic review identified DESH and quantitative DESH scoring among proposed imaging predictors of favorable shunt outcome, but also highlighted substantial inconsistency across studies.^13^ Similarly, two systematic reviews concluded that DESH alone does not reliably predict shunt responsiveness,^8,9^ and Carlsen et al. found no significant difference in DESH prevalence between shunt responders and nonresponders.^10^ Furthermore, two studies emphasized that absence of DESH should not be used to exclude patients from shunt surgery.^11,12^

Taken together, previous findings regarding the prognostic value of DESH remain inconsistent. Further evaluation of the DESH score in independent, representative iNPH cohorts using detailed and sensitive measures of postoperative outcome is therefore warranted. The aim of the present study was to investigate the association between preoperative DESH score and postoperative outcome, and to describe postoperative changes in DESH-related imaging findings after shunt surgery in a large consecutive cohort of patients with iNPH. The study was based on participants from the Gothenburg arm of the Prediction of Outcome in iNPH (POiNT) study,^14^ a prospective study designed to identify clinical and radiological markers predictive of shunt response in iNPH.

## Methods

### Patients

Between January 2014 and June 2016, 258 patients were investigated for suspected iNPH at the Hydrocephalus Unit, Sahlgrenska University Hospital, Gothenburg. These patients constituted the Gothenburg arm of the prospective Prediction of Outcome in iNPH (POiNT) study.^14^ The present study includes the first 101 consecutively enrolled patients with probable iNPH who underwent shunt surgery, had technically adequate preoperative MRI available for DESH assessment, and completed both pre- and postoperative clinical follow-up. The overall POiNT study design, patient flow, and reasons for exclusion have been described previously.^14^

All patients underwent neurological, physiotherapeutic, and neuropsychological examinations, as well as brain MRI. Previously diagnosed comorbidities, including hypertension, diabetes, and cardiovascular disease, were recorded during the preoperative evaluation.

Clinical symptom severity was graded using the Gothenburg iNPH scale introduced by Hellström.^15^ The iNPH scale provides a composite measure of symptom severity based on four domains: gait, balance, neuropsychology, and urinary continence. Scores range from 0 to 100, with higher scores indicating better function and less severe symptoms. Additional clinical assessments included the modified Rankin Scale (mRS), a measure of global functional disability ranging from 0 (no symptoms) to 5 (severe disability)^16^; the Mini-Mental State Examination (MMSE), a measure of global cognitive performance ranging from 0 to 30, with higher scores indicating better cognition^17^; and the Timed Up and Go test (TUG-t), a measure of gait and dynamic balance, reported as the time required to stand up from a chair, walk three meters, turn, walk back, and sit down.^18^ All clinical examinations and rating scales were performed preoperatively and repeated at postoperative follow-up. Complete data were not available for all outcome measures because some patients were unable or unwilling to complete individual assessments.

Patients fulfilled the criteria for probable iNPH according to the International Guidelines.^19^ Decisions regarding shunt surgery were made at multidisciplinary conferences based on the overall clinical presentation, medical history, neurological and functional assessments, and radiological findings. CSF tap testing was not routinely performed as a prerequisite for shunt surgery. In patients in whom the diagnosis remained uncertain, primarily because of indistinct or atypical symptoms, a CSF tap test or lumbar infusion test could be used as an additional diagnostic assessment to support the diagnosis.^14^ DESH score was not used as a criterion for surgical selection.

All patients received a programmable ventriculoperitoneal (*n* = 98) or ventriculoatrial shunt (*n* = 3) with a PS Medical Strata differential pressure valve and antisiphon device (Medtronic, Goleta, CA, USA), set at the standard opening pressure setting of 1.5 (medium setting). The choice of ventriculoperitoneal or ventriculoatrial shunt was based on the surgeons’ assessment of individual patients’ prerequisites. Patients underwent clinical and MRI follow-up after a median of 5 months (IQR 4–7 months) following surgery. At follow-up, all shunts were considered patent based on clinical assessment and CT or MRI findings; radionuclide shuntography or lumbar infusion testing was performed when shunt patency remained uncertain.

Before the postoperative follow-up, 6 patients had complications following shunt surgery. Three patients had major complications requiring new surgery due to distal mechanical shunt failures (2 had catheter misplacement and 1 had distal catheter obstruction). Three patients had subdural hematoma successfully treated with upregulation of the shunt valve opening pressure.

The study was approved by the Regional Ethics Committee (Dnr. 328-14, T439-15). Written informed consent was obtained from patients or their close relatives. The study was conducted in accordance with the Declaration of Helsinki.

### Outcome measures

Clinical improvement was primarily defined as an increase of at least five points on the iNPH scale score.^15^ Secondary measures of improvement included a decrease of at least one level on the mRS, an increase of at least three points on the MMSE, and a decrease of more than 10% in TUG-time.^4^

The primary endpoint was improvement in iNPH scale score at postoperative follow-up. Secondary outcome measures were postoperative changes in mRS, MMSE, and TUG-t. The association between preoperative DESH score and postoperative clinical improvement was evaluated according to the methodology described by Shinoda et al., with the iNPH scale score replacing the iNPH grading scale (iNPHGS).^4,20^

### MRI acquisition and image processing

MRI examinations were performed using a 1.5T Gyroscan Intera 9.1 system (Philips Medical Systems, Best, the Netherlands). The clinical MRI protocol included a 3D T1-weighted fast field echo sequence with the following parameters: TE 4.6 ms, TR 25 ms, flip angle 30°, no slice gap, in-plane resolution 1.2 × 1.2 mm, field of view 260 × 260 × 190 mm, acquisition matrix 260 × 259, and reconstructed voxel size 1 × 1 × 0.5 mm. Images were analyzed using RadiAnt DICOM Viewer (Medixant, Version 4.6.5)^21^ or 3D Slicer (Version 4.11.20210226; https://www.slicer.org/).^22^ Slice orientation was manually re-angled when necessary to enable standardized DESH score assessment.

### DESH score assessment

Pre- and postoperative DESH scores were independently assessed on T1-weighted images by one senior neurologist (M.T.), one senior neurosurgeon (D.F.), and one neuroradiology fellow (K.S.), all blinded to the clinical outcome of the shunt surgery. DESH scoring was performed in accordance with the methodology described by Shinoda et al.^4^ using strict predefined criteria for all score components. In cases of disagreement between the three readers, a senior neuroradiologist (D.Z.) determined the final DESH score.

The *DESH score* comprised five radiological features, each graded from 0 to 2, yielding a maximum score of 10 points: ventriculomegaly, Sylvian fissure dilation, tight high convexity (THC), acute callosal angle, and focal sulcal dilation (Table 1).^4^

**Table 1.** Summary of DESH score.

DESH feature	Grade	Definition
Ventriculomegaly	0	Normal ventricular size (Evans’ index <0.30)
	1	Mild ventriculomegaly (Evans’ index 0.30–0.35)
	2	Marked ventriculomegaly (Evans’ index >0.35)
Sylvian fissure dilation	0	Normal or narrow Sylvian fissures
	1	Mild or unilateral dilation
	2	Bilateral dilation
Tight high convexity	0	Normal or widened convexity sulci
	1	Mild compression
	2	Pronounced compression
Callosal angle	0	Obtuse angle (>100°)
	1	Intermediate angle (90°–100°)
	2	Acute angle (<90°)
Focal sulcal dilation	0	No focally enlarged sulci
	1	Few focally enlarged sulci
	2	Multiple focally enlarged sulci

The DESH score includes five radiological features associated with idiopathic normal pressure hydrocephalus (iNPH), each graded from 0 to 2, with higher scores indicating more pronounced DESH-related changes.

Abbreviations: DESH, disproportionately enlarged subarachnoid-space hydrocephalus.

*Ventriculomegaly* was assessed on transverse images parallel to the anterior commissure–posterior commissure (AC-PC) plane at the level of maximal frontal horn width of the lateral ventricles. Evans’ index (EI) was calculated as the ratio between the maximal frontal horn diameter and the maximal inner skull diameter in the same section.^23^ Ventriculomegaly was graded as normal (EI <0.30), slight dilation (EI 0.30–0.35), or definite dilation (EI >0.35).^4^

*Sylvian fissure dilation* was evaluated on coronal images reconstructed parallel to the brainstem at the level of the central brainstem and graded as normal or narrow (0), slight or unilateral dilation (1), or bilateral dilation (2).^24,25^

*Tight high convexity* was evaluated on transverse and coronal images perpendicular to the AC-PC plane and graded as normal or widened convexity sulci (0), slight compression (1), or definitive compression (2).^26^

*The callosal angle* was measured as the angle between the lateral ventricles on coronal images perpendicular to the AC-PC plane at the level of the PC and graded as obtuse (>100°), intermediate (90°–100°), or acute (<90°).^27^

*Focal sulcal dilation* was defined as focal enlargement of cortical sulci without generalized cortical atrophy and without continuity with the Sylvian fissure. Sulci were required to have a rounded or oval appearance and were graded as absent (0), some focally enlarged sulci (1), or multiple focally enlarged sulci (2).^28,29^

### Statistical analysis

Normality of variable distributions was assessed using graphical inspections (histograms with overlaid normal curves). Distributions were considered normally distributed, and parametric statistical methods were therefore applied. Group comparisons between independent groups were performed using Student’s *t*-test, and paired t-tests were used to evaluate changes between preoperative and postoperative assessments. Associations between continuous variables were analyzed using Pearson’s correlation coefficient. To assess whether preoperative DESH score was independently associated with postoperative iNPH scale score, a multivariable linear regression analysis was performed with postoperative iNPH scale score as the dependent variable. Preoperative DESH score was entered together with baseline iNPH scale score, age at surgery, and disease duration as covariates. Results are presented as unstandardized regression coefficients (B) with standard errors (SEs), 95% confidence intervals (CIs), and p values. A sensitivity analysis additionally included diabetes, hypertension, and cardiovascular disease. Postoperative changes in total DESH score and the individual DESH components were compared between patients with and without clinically meaningful improvement on the iNPH scale using Mann–Whitney *U* tests. P values for the five individual DESH component comparisons were adjusted for multiple testing using the Holm method. Interobserver agreement among the three independent readers was assessed for the total DESH score and each individual DESH component using a two-way random-effects, absolute-agreement, single-measure intraclass correlation coefficient [ICC(2,1)], with 95% confidence intervals. Agreement was evaluated separately for preoperative and postoperative assessments. Categorical variables are presented as counts and percentages, and continuous variables as mean ± standard deviation (SD). Analyses were based on available data for each outcome measure; missing values were not imputed. All significance tests were two-tailed, and alpha was set to 0.05.

Statistical analyses were performed using IBM SPSS Statistics for Windows, version 28.0.1.1 (IBM Corp., Armonk, NY, USA).

## Results

### Patient population

A total of 101 patients with shunt-treated iNPH were included. Baseline demographic and clinical data are presented in Table 2. All patients had an EI >0.30. The mean DESH score was 6.3 ± 1.7 (range 2–10), with an approximately normal distribution (Figure 1).

**Table 2.** Baseline characteristics of the study population.

Characteristic	*n*	Value
*Demographics*		
Age, years	101	74.4 ± 6.5 (59–91)
Male sex, *n* (%)	101	68 (67)
Disease duration, months	101	40 ± 29 (5–120)
Diabetes, *n* (%)	101	31 (31)
Hypertension, *n* (%)	101	63 (62)
Cardiovascular disease, *n* (%)	101	33 (33)

Values are presented as mean ± standard deviation (SD) with range in parentheses for continuous variables, and as number (%) for categorical variables.

**Figure 1.**
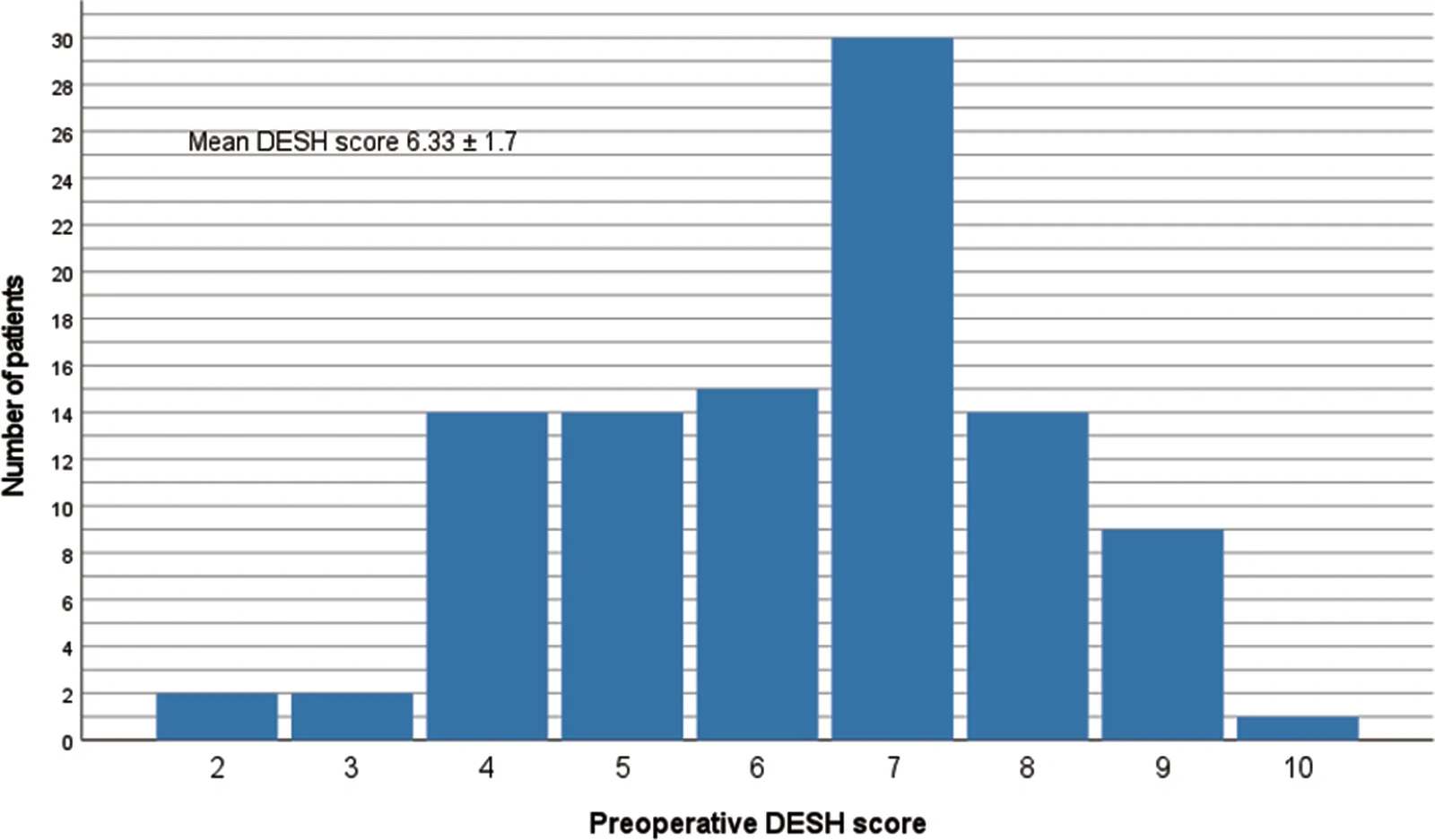
Distribution of preoperative DESH scores.

### Clinical response to shunt surgery

At follow-up, 70% of patients met the predefined primary outcome of clinically meaningful improvement, defined as an increase of ≥5 points on the iNPH scale score. Among the secondary outcomes, 37% improved by at least one mRS level, 14% improved by ≥3 points on the MMSE, and 69% showed a >10% reduction in TUG-t. Pre- and postoperative values for all clinical outcome measures are presented in Table 3.

**Table 3.** Clinical outcomes before and after shunt surgery.

Variable	*n*	Before surgery, mean ± SD	After surgery, mean ± SD	*p* value
iNPH scale score	101	50.8 ± 19.8	63.3 ± 20.0	**<0.001**
mRS score	81	3.61 ± 0.75	3.28 ± 0.90	**<0.001**
MMSE score	98	25.0 ± 4.0	25.4 ± 4.0	0.177
TUG-t, s	83	23.9 ± 14.1	16.5 ± 8.0	**<0.001**

Values are presented as mean ± standard deviation (SD). The number of patients included in each analysis is shown in the n column and reflects available paired pre- and postoperative data for the respective outcome measure. P values were calculated using paired t-tests. Bold values indicate statistically significant p values (p < 0.05).

Abbreviations: iNPH, idiopathic normal pressure hydrocephalus; MMSE, Mini-Mental State Examination; mRS, modified Rankin Scale; s, seconds; SD, standard deviation; TUG-t, Timed Up and Go test.

### Association between preoperative DESH score and clinical response

The preoperative DESH score showed a weak inverse correlation with postoperative change in iNPH scale score; however, the association was not statistically significant (Pearson *r* = −0.123, *p* = .219) (Figure 2).

**Figure 2.**
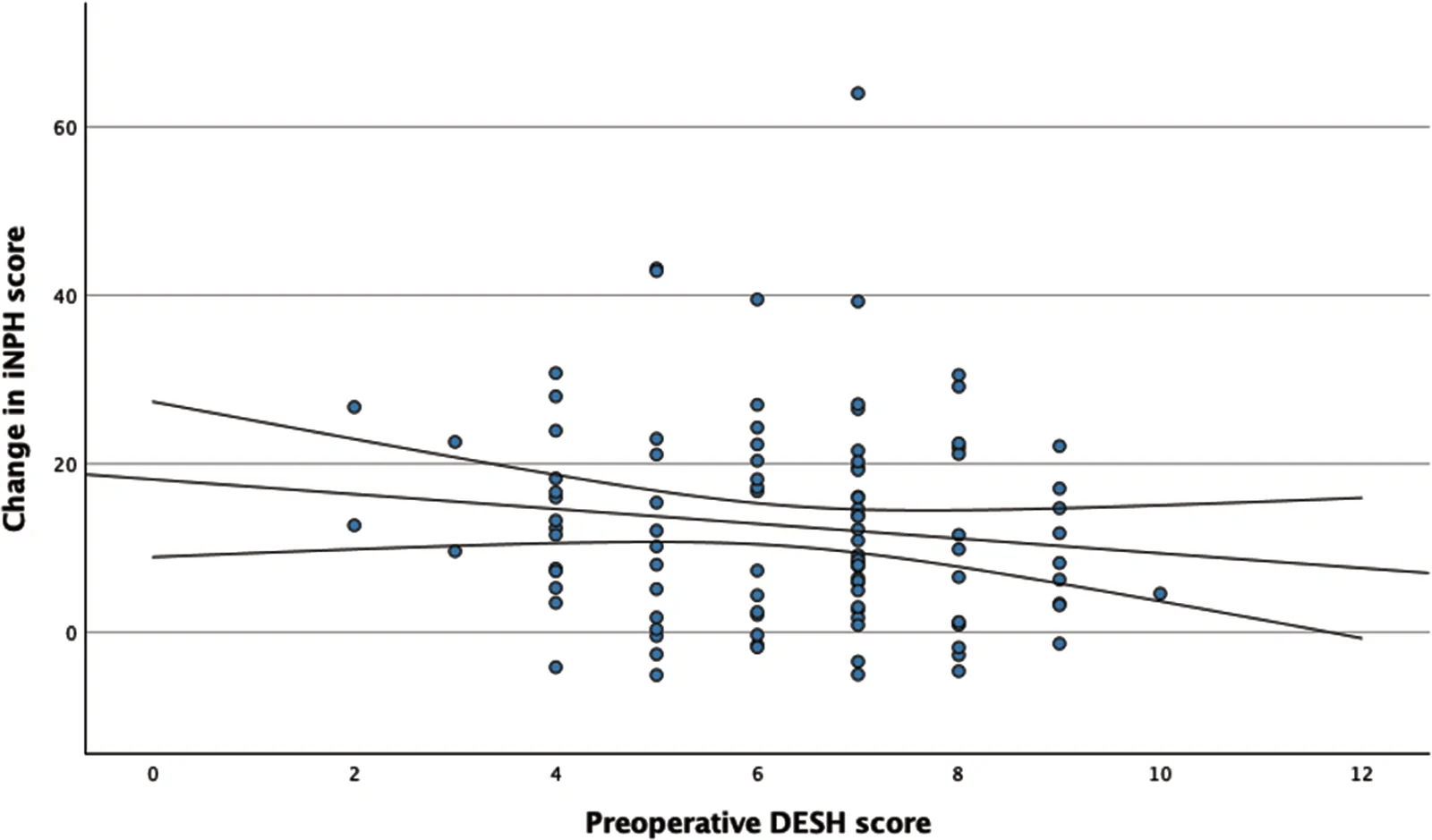
Association between preoperative DESH score and postoperative change in iNPH scale score. The solid line represents the fitted linear regression line, and the curved lines represent the 95% confidence interval. A weak inverse correlation was observed, although the association was not statistically significant (Pearson *r* = −0.123, *p* = .219).

In multivariable linear regression analysis, preoperative DESH score was not independently associated with postoperative iNPH scale score after adjustment for baseline iNPH scale score, age at surgery, and disease duration (Table 4). Baseline iNPH scale score and age were independently associated with postoperative iNPH scale score, whereas disease duration was not. The association between preoperative DESH score and postoperative iNPH scale score remained non-significant in the sensitivity analysis additionally adjusting for diabetes, hypertension, and cardiovascular disease (Supplemental Table 1).

**Table 4.** Multivariable linear regression analysis of postoperative iNPH scale score.

Variable	B	SE(B)	95% CI	*p* value
Preoperative DESH score, per point	−0.57	0.69	−1.95 to 0.80	0.409
Age at surgery, per year	−0.49	0.19	−0.86 to −0.12	**0.011**
Baseline iNPH scale score, per point	0.81	0.06	0.69 to 0.92	**<0.001**
Disease duration, per month	0.02	0.04	−0.06 to 0.11	0.594

The dependent variable was postoperative iNPH scale score. The multivariable linear regression analysis included 99 patients with complete data. Regression coefficients are adjusted for all variables included in the model. The model explained 69.0% of the variance in postoperative iNPH scale score (R^2^ = 0.690; adjusted R^2^ = 0.677). Bold values indicate statistically significant p values (p < 0.05).

Abbreviations: B, unstandardized regression coefficient; CI, confidence interval; DESH, disproportionately enlarged subarachnoid-space hydrocephalus; iNPH, idiopathic normal pressure hydrocephalus; SE(B), standard error of the unstandardized regression coefficient.

Preoperative DESH scores did not differ significantly between patients with and without postoperative improvement according to the primary outcome (iNPH scale score) or any of the secondary outcomes (mRS, MMSE, and TUG-t) (Table 5).

**Table 5.** Preoperative DESH scores in patients with and without postoperative improvement according to primary and secondary outcome measures.

Outcome measure	Improved, *n*	DESH score, mean ± SD	Not improved, *n*	DESH score, mean ± SD	*p* value
iNPH scale score	71	6.1 ± 1.7	30	6.8 ± 1.5	0.093
mRS	30	6.2 ± 1.7	51	6.7 ± 1.5	0.185
MMSE	14	6.6 ± 2.5	84	6.3 ± 1.5	0.568
TUG-t	57	6.4 ± 1.7	26	6.6 ± 1.7	0.604

Values are presented as mean ± standard deviation (SD). Improvement was defined as an increase of ≥5 points on the iNPH scale score, a decrease of ≥1 point on the modified Rankin Scale (mRS), an increase of ≥3 points on the Mini-Mental State Examination (MMSE), and a reduction of >10% in Timed Up and Go test (TUG-t) time. P values were calculated using Student’s t-test.

Abbreviations: DESH, disproportionately enlarged subarachnoid-space hydrocephalus; iNPH, idiopathic normal pressure hydrocephalus; MMSE, Mini-Mental State Examination; mRS, modified Rankin Scale; SD, standard deviation; TUG-t, Timed Up and Go test.

### Radiological changes after shunt surgery

DESH-related imaging markers changed significantly after shunt surgery (Table 6). DESH score and Evans’ index decreased, whereas callosal angle increased. When stratified according to the primary clinical outcome, changes in total DESH score and the individual DESH components did not differ significantly between patients with and without clinically meaningful improvement on the iNPH scale (Table 7).

**Table 6.** Changes in DESH-related radiological markers between preoperative and postoperative MRI examinations.

Variable	Preoperative mean (SD)	Postoperative mean (SD)	*p* value
DESH score	6.3 (1.7)	4.6 (1.8)	**<0.001**
Evans’ index	0.39 (0.04)	0.37 (0.05)	**<0.001**
Callosal angle, degrees	67.3 (14.8)	80.8 (19.8)	**<0.001**

Values are presented as mean (SD). P values were calculated using paired t-tests comparing preoperative and postoperative measurements. Bold values indicate statistically significant p values (p < 0.05).

Abbreviations: DESH, disproportionately enlarged subarachnoid-space hydrocephalus; SD, standard deviation.

**Table 7.** Pre- and postoperative DESH score and individual components according to improvement in iNPH scale score.

DESH measure	Grade	Preop, all	Preop, improved	Preop, not improved	Postop, all	Postop, improved	Postop, not improved	*p* value
Total DESH score, mean ± SD	—	6.3 ± 1.7	6.1 ± 1.8	6.8 ± 1.5	4.6 ± 1.8	4.5 ± 1.8	4.8 ± 1.8	0.888^a^
Sylvian fissure dilation, *n* (%)	0	19 (19)	15 (21)	4 (13)	35 (35)	26 (37)	9 (30)	1.000
	1	65 (64)	45 (63)	20 (67)	57 (56)	40 (56)	17 (57)	​
	2	17 (17)	11 (15)	6 (20)	9 (9)	5 (7)	4 (13)	​
Tight high convexity, *n* (%)	0	27 (27)	21 (30)	6 (20)	76 (75)	57 (80)	19 (63)	1.000
	1	50 (50)	34 (48)	16 (53)	25 (25)	14 (20)	11 (37)	​
	2	24 (24)	16 (23)	8 (27)	0 (0)	0 (0)	0 (0)	​
Focal sulcal dilation, *n* (%)	0	47 (47)	35 (49)	12 (40)	71 (70)	50 (70)	21 (70)	1.000
	1	38 (38)	27 (38)	11 (37)	19 (19)	14 (20)	5 (17)	​
	2	16 (16)	9 (13)	7 (23)	11 (11)	7 (10)	4 (13)	​
Ventriculomegaly, *n* (%)	0	0 (0)	0 (0)	0 (0)	6 (6)	4 (6)	2 (7)	1.000
	1	21 (21)	15 (21)	6 (20)	26 (26)	21 (30)	5 (17)	​
	2	80 (79)	56 (79)	24 (80)	69 (68)	46 (65)	23 (77)	​
Callosal angle, *n* (%)	0	5 (5)	5 (7)	0 (0)	18 (18)	11 (15)	7 (23)	1.000
	1	1 (1)	1 (1)	0 (0)	11 (11)	9 (13)	2 (7)	​
	2	95 (94)	65 (92)	30 (100)	72 (71)	51 (72)	21 (70)	​

Values are presented as mean ± standard deviation (SD) for total DESH score and number (%) for individual DESH components. Improvement was defined as an increase of ≥5 points on the iNPH scale score. Individual DESH components were graded from 0 to 2 according to the criteria presented in Table 1. P values compare the pre- to postoperative change between improved and non-improved patients. P values for the five individual DESH components were adjusted for multiple comparisons using the Holm method.

Abbreviations: DESH, disproportionately enlarged subarachnoid-space hydrocephalus; iNPH, idiopathic normal pressure hydrocephalus; SD, standard deviation.

^a^The p value for total DESH score was not included in the multiple-comparison correction.

Interobserver agreement for the total DESH score was 0.717 (95% CI 0.632–0.789) preoperatively and 0.649 (95% CI 0.508–0.755) postoperatively. Agreement for the individual DESH components varied, with ICCs ranging from 0.572 to 0.708 preoperatively and from 0.317 to 0.790 postoperatively (Supplemental Table 2).

## Discussion

In this prospective cohort study of shunt-treated patients with iNPH, preoperative DESH score was not associated with postoperative improvement in the iNPH scale score, mRS, MMSE, or TUG-t outcomes. Although a weak inverse correlation was observed between DESH score and postoperative change in iNPH scale score, this relationship was not statistically significant. In multivariable analysis, preoperative DESH score was not independently associated with postoperative iNPH scale score after adjustment for baseline iNPH scale score, age at surgery, and disease duration. This finding remained consistent after additional adjustment for diabetes, hypertension, and cardiovascular disease. Likewise, preoperative DESH scores did not differ between patients with and without postoperative improvement across any evaluated outcome measure. DESH-related imaging markers, however, changed significantly after shunt surgery.

These findings differ from the original study by Shinoda et al., which reported associations between higher DESH scores and greater postoperative improvement in mRS, iNPHGS, MMSE, TMT-A, and TUG-t outcomes.^4^ Several subsequent studies have similarly reported associations between higher DESH scores and favorable postoperative outcome,^5–7^ whereas others, including systematic reviews, have found insufficient evidence that DESH alone reliably predicts shunt responsiveness.^8,9,11,12^

Several methodological factors may contribute to these conflicting findings. Patient selection is likely important. In previous studies, inclusion or surgical selection depended on features such as DESH, a positive CSF tap test, or increased resistance to CSF outflow,^4,7^ potentially enriching study populations with patients more likely to respond to shunting. In our cohort, CSF tap testing was not routinely required for surgical selection but was used selectively, together with lumbar infusion testing, when the diagnosis was uncertain. Apart from ventriculomegaly, DESH-related imaging features were not required for inclusion, and the DESH score was evaluated independently as a prognostic marker. This approach may have reduced selection bias and allowed assessment across a broader and more clinically representative spectrum of shunt-treated iNPH patients.

Differences in outcome definitions may also have contributed. In a recent validation study, the postoperative outcome was assessed using a composite shunt response score based on changes in the gait and continence domains of the iNPH scale together with MMSE, with a score of ≥1 defined as improvement.^7^ In contrast, our study used a predefined threshold of ≥5 points on the Gothenburg iNPH scale score to define clinically meaningful improvement. Different outcome measures and responder thresholds may influence observed associations between DESH score and shunt response. Less stringent responder definitions may increase sensitivity for detecting weak associations between imaging markers and outcome but may also classify patients with minimal clinical improvement as responders. Furthermore, another study reporting associations between higher DESH scores and postoperative improvement relied partly on subjective outcome assessments,^6^ whereas our study used predefined objective clinical outcome measures.

Our findings support the interpretation of DESH as a supportive imaging feature of iNPH rather than a robust independent predictor of shunt response. Previous studies have shown substantial heterogeneity in DESH findings, with many patients with clinically diagnosed iNPH exhibiting incomplete or absent DESH patterns.^11,12,25,30,31^ In two Swedish population-based cohorts, complete DESH was present in only 7.5% and 17% of individuals with radiological signs of iNPH.^1,32^ Moreover, patients without DESH may still benefit from shunt surgery.^11,12^ Consistent with this, 70% of patients in our cohort showed clinically meaningful improvement despite the lack of a significant association between DESH score and outcome. In clinical practice, DESH findings should therefore be interpreted as part of the overall clinical and radiological assessment rather than as an isolated criterion for surgical eligibility. Future predictive models may benefit from combining DESH-related imaging features with other clinical, imaging, CSF-dynamic, or biomarker-based measures to better identify patients likely to benefit from shunt surgery.

DESH-related imaging markers changed significantly after shunt surgery, with reductions in DESH score and Evans’ index and an increase in callosal angle. However, the evolution of the individual DESH components did not differ significantly between patients with and without clinically meaningful improvement on the iNPH scale. These findings suggest that DESH-related morphological changes are at least partly reversible following CSF diversion but are not closely coupled to clinical response, supporting the interpretation that DESH may primarily reflect disease morphology rather than treatment responsiveness.

The present study has several strengths. Patients were prospectively and consecutively included from a tertiary hydrocephalus center, and standardized pre- and postoperative clinical assessments were used. DESH scoring was performed independently by multiple raters who were blinded to postoperative clinical outcomes, with neuroradiological adjudication in cases of disagreement, reducing the risk of observer bias. In addition, several clinically relevant outcome measures were evaluated, including gait, cognition, disability, and overall iNPH symptom severity.

Several limitations should also be acknowledged. First, this was a single-center study with a moderate sample size, which may limit statistical power and generalizability. Although no significant associations between DESH score and postoperative outcomes were identified, the possibility of a type II error cannot be excluded, and smaller associations may therefore have gone undetected. Second, although data collection was prospective, complete paired data were not available for all outcome measures because some patients were unable or unwilling to complete individual assessments. This reduced the sample size for some secondary outcome analyses and may have reduced their statistical precision. Third, postoperative follow-up was relatively short, and longer-term outcomes were not assessed. As both clinical and radiological changes may evolve over time after shunt surgery, longer follow-up could potentially reveal associations between DESH-related imaging findings and clinical outcome that were not apparent at the present follow-up. Fourth, DESH scoring also remains partly subjective despite standardized grading criteria and neuroradiological adjudication. Interobserver agreement was higher for the total DESH score than for some individual components, with particularly lower agreement for postoperative tight high convexity and focal sulcal dilation ratings. This variability highlights the potential for interobserver differences in the assessment of individual DESH features. However, all assessments were performed independently by three blinded readers, and in cases of disagreement, a senior neuroradiologist adjudicated the final DESH score. This standardized procedure reduced the potential impact of interobserver variability on the final ratings used in the analyses. Fifth, lumbar tap testing was performed selectively rather than systematically, and detailed tap-test response data were not available for the present analysis. We were therefore unable to evaluate whether the association between DESH score and postoperative outcome differed according to tap-test response. Sixth, the use of a medium shunt valve setting of 1.5 in all patients, including both VP and VA shunts and independent of patients’ weight and height, may limit the efficacy of treatment in some patients compared to lower valve settings. This design is based on our previous long-term clinical experience and used to avoid introduction of bias related to different valve settings across groups of patients. Finally, other potentially relevant radiological, physiological, or biomarker-based predictors were not included in the present analyses.

## Conclusions

In this prospective cohort of shunt-treated patients with iNPH, the preoperative DESH score was not associated with postoperative improvement in clinical outcome measures. Although DESH may represent a supportive imaging feature of iNPH, our findings suggest that the DESH score alone has limited value as a predictor of shunt responsiveness. Importantly, absence of pronounced DESH findings should not be used to exclude patients from shunt surgery.

## Supplemental material

Supplemental material - Radiological DESH score cannot predict outcome after shunt surgery: A Swedish prospective follow-up study Supplemental material for Radiological DESH score cannot predict outcome after shunt surgery: A Swedish prospective follow-up study by Clara Constantinescu, M. Emgård, K. Sundström, D. Farahmand, M. Tullberg, D. Ziegelitz, and P. Hellström in The Neuroradiology Journal.

## Data Availability

The datasets generated and/or analyzed during the current study are not publicly available due to ethical and privacy restrictions but are available from the corresponding author on reasonable request.*
