# Supplementary material for: Radiological DESH score cannot predict outcome after shunt surgery: A Swedish prospective follow-up study
Source: Neuroradiol J. 2026 Oct 7:19714009261494951. Online ahead of print. doi: 10.1177/19714009261494951 (PMC13646207; doi:10.1177/19714009261494951)
Supplement: Supplementary file 1 [file sj-pdf-1-neu-10.1177_19714009261494951.pdf]

Appendix

Supplementary Table 1. Sensitivity analysis of postoperative iNPH scale score

Variable	B	SE(B)	95% CI	p value
**Preoperative DESH score, per point**	−0.73	0.69	−2.11 to 0.64	0.293
**Age at surgery, per year**	−0.41	0.20	−0.80 to −0.02	0.038
**Baseline iNPH scale score, per point**	0.80	0.06	0.69 to 0.92	<0.001
**Disease duration, per month**	0.02	0.04	−0.06 to 0.10	0.645
**Diabetes**	−0.99	2.63	−6.21 to 4.23	0.707
**Hypertension**	2.66	2.51	−2.32 to 7.64	0.291
**Cardiovascular disease**	−4.70	2.56	−9.79 to 0.38	0.069

The dependent variable was postoperative iNPH scale score. The sensitivity analysis included 99 patients with complete data and extended the primary model by additionally including diabetes, hypertension, and cardiovascular disease. Regression coefficients are adjusted for all variables included in the model. The model explained 70.3% of the variance in postoperative iNPH scale score ($R^2 = 0.703$; adjusted $R^2 = 0.680$). B, unstandardized regression coefficient; CI, confidence interval; DESH, disproportionately enlarged subarachnoid-space hydrocephalus; iNPH, idiopathic normal pressure hydrocephalus; SE(B), standard error of the unstandardized regression coefficient.

Supplementary Table 2. Interobserver agreement for DESH score and individual DESH components

DESH measure	Preoperative n	Preoperative ICC (95% CI)	Postoperative n	Postoperative ICC (95% CI)
**Sylvian fissure dilation**	100	0.610 (0.493–0.710)	101	0.542 (0.326–0.693)
**Tight high convexity**	100	0.599 (0.489–0.696)	101	0.317 (0.195–0.444)
**Focal sulcal dilation**	100	0.572 (0.463–0.671)	100	0.324 (0.199–0.452)
**Ventriculomegaly**	100	0.708 (0.611–0.787)	101	0.790 (0.709–0.851)
**Callosal angle grade**	100	0.647 (0.548–0.734)	101	0.664 (0.516–0.769)
**Total DESH score**	100	0.717 (0.632–0.789)	100	0.649 (0.508–0.755)

Interobserver agreement among the three independent readers was assessed using a two-way random-effects, absolute-agreement, single-measure intraclass correlation coefficient [ICC(2,1)]. ICCs with 95% confidence intervals (CIs) are presented separately for preoperative and postoperative assessments. The number of observations reflects patients with complete ratings from all three readers for the respective assessment. DESH, disproportionately enlarged subarachnoid-space hydrocephalus; ICC, intraclass correlation coefficient.
